# Innovative methods for observing and changing complex health behaviors: four propositions

**DOI:** 10.1093/tbm/ibaa026

**Published:** 2020-05-18

**Authors:** Guillaume Chevance, Olga Perski, Eric B Hekler

**Affiliations:** 1 Center for Wireless and Population Health Systems, University of California, San Diego, La Jolla, CA, USA; 2 Exercise and Physical Activity Resource Center, University of California, San Diego, La Jolla, CA, USA; 3 Family Medicine and Public Health, University of California, San Diego, La Jolla, CA, USA; 4 Department of Behavioural Science and Health, University College London, Torrington Place, London, UK

**Keywords:** Precision health, Idiographic, Ecological momentary assessment, Computational models, Adaptive interventions

## Abstract

Precision health initiatives aim to progressively move from traditional, group-level approaches to health diagnostics and treatments toward ones that are individualized, contextualized, and timely. This article aims to provide an overview of key methods and approaches that can help facilitate this transition in the health behavior change domain. This article is a narrative review of the methods used to observe and change complex health behaviors. On the basis of the available literature, we argue that health behavior change researchers should progressively transition from (i) low- to high-resolution behavioral assessments, (ii) group-only to group- and individual-level statistical inference, (iii) narrative theoretical models to dynamic computational models, and (iv) static to adaptive and continuous tuning interventions. Rather than providing an exhaustive and technical presentation of each method and approach, this article articulates why and how researchers interested in health behavior change can apply these innovative methods. Practical examples contributing to these efforts are presented. If successfully adopted and implemented, the four propositions in this article have the potential to greatly improve our public health and behavior change practices in the near future.

## ACCOUNTING FOR THE INHERENT COMPLEXITY OF HEALTH BEHAVIOR CHANGE

Precision medicine [1] and precision health [2] (i.e., diagnostics and treatment strategies that take individual variability into account) have been greatly expanded in the past years through a variety of methods and computational techniques, such as genome sequencing, big data, and wearable technology. These initiatives aim to progressively move beyond approaches focused on average patient responses toward ones that are individualized, contextualized, and timely [2]. In this article, we aim to provide an overview of key methods and approaches that can help facilitate the transition to precision initiatives in the health behavior change domain.

Health behaviors include any activities undertaken by individuals for the purpose of maintaining, enhancing, or protecting their health [3], such as regularly practicing physical activity, eating healthily, limiting alcohol consumption, and abstaining from tobacco smoking. Despite the well-established effects of such behaviors on health and important prevention efforts in the past decades, the majority of people in high income countries experience difficulties in adopting and maintaining health behaviors in the long run [4,5]. In the USA and Europe, only a small proportion of people (20–30%) report meeting the national physical activity guidelines [4]. In the same regions of the world, 10–30% are cigarette smokers [6] and 30% of adolescents in the USA report consuming at least one sugar-sweetened beverage per day [7].

In contrast with low-occurrence behaviors (e.g., cancer screening and vaccination uptake), repeated-occurrence behaviors (e.g. physical activity, alcohol reduction) have to be performed (or avoided) on a regular basis over the entire lifespan, across different contexts [8]. Repeated-occurrence behaviors therefore tend to be dynamic, multi-factorial, and idiosyncratic; research shows that behaviors such as physical activity and sleep [9], eating behaviors [10,11], and smoking lapses [11] vary from day to day at the individual level [12,13] in response to a dynamic interplay of intra-individual (e.g., motivation), inter-individual (i.e., social support), and environmental/contextual factors (i.e., weather) [14]. As these behaviors are dynamic and manifest idiosyncratically (i.e., differently from one person to another), one specific influence (e.g. social support, weather) or intervention may be more or less informative/effective for different individuals [15].

In this article, our central argument is that, besides theoretical innovations [16–19], the efficient promotion of health behaviors requires the use of innovative methods which take account of the inherent complexity of health behavior change. By complexity, we refer to the dynamic, multi-factorial, and idiosyncratic nature of health behavior change. The aim of this article was to articulate a set of innovative methods and approaches that might help to better address the complexity of health behavior change. We wish to provide a comprehensive overview of why and how researchers can apply these innovative methods and how, in concert, they will enable a form of precision behavioral science. Specifically, we argue the following: health behavior change researchers should progressively transition from (i) low- to high-resolution behavioral assessments, (ii) group-only to group- and individual-level statistical inference, (iii) narrative theoretical models to dynamic computational models, and (iv) static to adaptive and continuous tuning interventions (see Table 1).

**Table 1:** Summary of the propositions made in the article and their key benefits.

Propositions	Key benefits
1. From low- to high-resolution behavioral assessments	– Enables researchers to capture the potential dynamic nature of behaviors and their determinants, including their relative variability or stability over time. – Enables the exploration of questions relating to spatial and temporal synchronicity of behaviors and their determinants. – Reduces the likelihood of erroneous conclusions due to the use of an inappropriate sampling frequency.
2. From group-only to group- and individual-level statistical inference	– Results in rapid, efficient, and cost-efficient learning. – Directly benefits the unit who provided the data (e.g., the patient). – Enables the exploration of individual differences, which may be more or less relevant for different phenomena. – Prevents potential fallacies of unfounded group-to-individual generalizability.
3. From narrative models to dynamic computational models	– Enables formalized and more precise behavioral hypotheses, taking temporal, contextual, and individual aspects into account. – Produces transparent theories that can be refuted, revised, and/or extended. -– Enables the testing of hypotheses that would otherwise be difficult to explore due to practical limitations (e.g., phenomena that unfold across the lifespan).
4. From static to adaptive and continuous tuning interventions	– Enables the provision of the right type and intensity of support to individuals at the right time. – Continuous tuning interventions learn over time what type of support works directly from the unit who provided the data. – Enables the provision of highly tailored support via digital tools (or a combination of computer and human support) which is presently only offered by “health coaches.”

### From low- to high-resolution behavioral assessments

**Figure 1:**
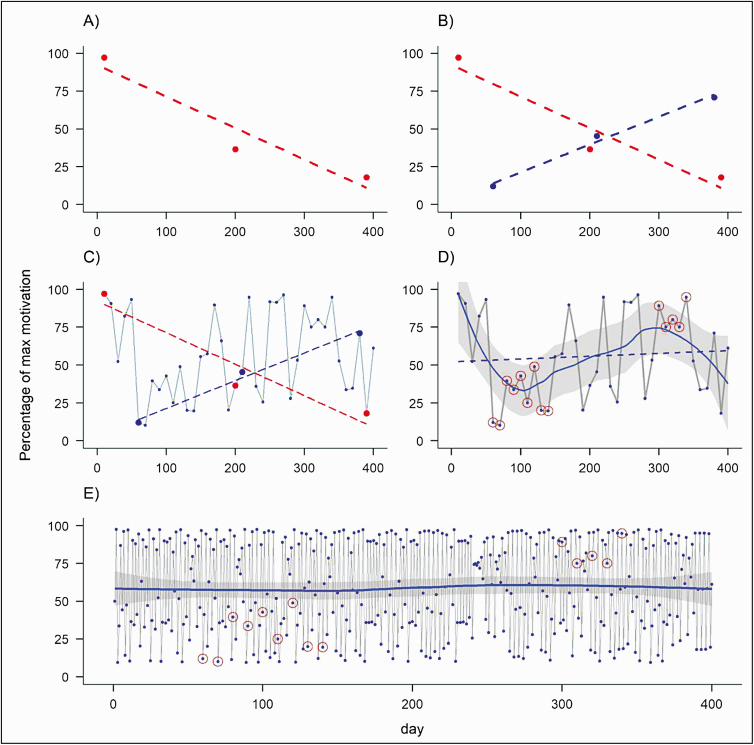
Simulated percentages of a person’s level of motivation (*y*-axis) modeled over time (*x*-axis); (A) measurement of motivation across three time points, representing conventional intervention evaluation at baseline, post-intervention, and at a longer-term follow-up; (B) measurement of motivation on different days compared with (A) but maintaining the same measurement frequency; (C) measurements at a higher sampling frequency (40 time points instead of three); (D) linear regression line (dashed) and LOESS regression line (solid), fitted to the measurements in (C); (E) measurements at a higher sampling frequency (400 time points instead of 40), revealing a process of “deterministic chaos.” Figure courtesy of Matti Heino, University of Helsinki, see [27].

The health behavior change literature has been dominated by a low-resolution measurement paradigm, illustrated by the high volume of studies adopting cross-sectional, prospective, longitudinal (nonintensive), and pre- and postintervention research designs [20]. In the past decades, greater accessibility to technologies such as smartphones and wearable devices (e.g., smart watches) has facilitated the real-time (or near real-time) assessment of health behaviors and their influences/outcomes in daily life [21,22]. Studies incorporating repeated assessments have highlighted the dynamic nature of health behaviors and their influences, illustrated at different time scales [9,23,24]. In addition to conceptual questions that can only be addressed through high-resolution data, such as the variability/instability of a particular process or questions of temporal and spatial synchronicity [25], failing to address the dynamics of health behaviors (e.g., by observing a phenomenon at the wrong temporal scale) can lead to incomplete or sometimes even erroneous conclusions [26]. For example, results from a randomized controlled trial in which group differences in alcohol consumption post-intervention were measured at 30 different time points demonstrated that focusing on the commonly used 1- and 6-month follow-up assessments might lead to erroneous conclusions about the effectiveness of an intervention [23]. The importance of observing a phenomenon at the right temporal frequency is graphically illustrated in Fig. 1 (for an empirical example, see [28]).

Empirically, one way to avoid the issue highlighted above is to measure the variables/phenomena of interest at the highest temporal resolution possible given, for example, physical constraints of measurement devices. Starting with an initially high resolution (e.g., measuring physical activity continuously over the course of a trial), one can then identify when meaningful variance occurs (Fig. 1). Theoretically, when selecting the temporal resolution, it is useful to draw on the literature in terms of the extent to which and over what time frame the variables of interest are expected to display meaningful variation [20]. Using that, one can then seek to establish a measurement protocol that does not violate the “Nyquist Principle” (for a definition, see [29]). Specifically, the Nyquist Principle postulates that, for any given phenomenon, the sampling rate needs to be twice the degree to which meaningful variance and change is observed. For example, if theory postulates that meaningful variation in motivation will occur every 30 min, then sampling should, ideally, occur at least every 15 min. If neither prior empirical work nor theory is a useful guide, then a practical approach is more appropriate. Namely, one should select the highest resolution that is practically feasible and work backwards from there.

In practice, behavioral monitoring can be done through passive detection (e.g., recording the number of steps on any given day via an accelerometer or amount of rain via GPS-derived information linked with weather sensor data) or actively by asking participants to answer a series of brief questions or complete short tests. At present, the most widely used real-time monitoring method is the “experience sampling method” (ESM) (also known as “ecological momentary assessments”; EMAs), the “measurement-burst design,” and “digital phenotyping.” EMAs and ESM involve the measurement of relevant psychological and behavioral phenomena in or near real-time [30]. Measurement-burst designs involve widely spaced successions of short EMAs/ESM periods, repeated over longer time periods [31] (e.g., 1-week, 3 times per year). Digital phenotyping involves the use of sensors and digital traces (e.g., information gathered passively via the use of digital tools, such as free-form text, app and social media interactions) to infer psychological and behavioral constructs [32].

### From group-only to group- and individual-level statistical inference

Measuring behaviors and their influences at a high resolution has another key implication: the sample size becomes the function of the total number of *observations per individual*, as opposed to the total *number of individuals*. This allows for individual statistical modeling [33,34] (i.e., building a separate statistical model for each individual). This approach to statistical modeling is also termed “idiographic,” and is distinct from the traditional, “nomothetic” approach, which relies on the pooling of data from a group of participants [35].

**Figure 2:**
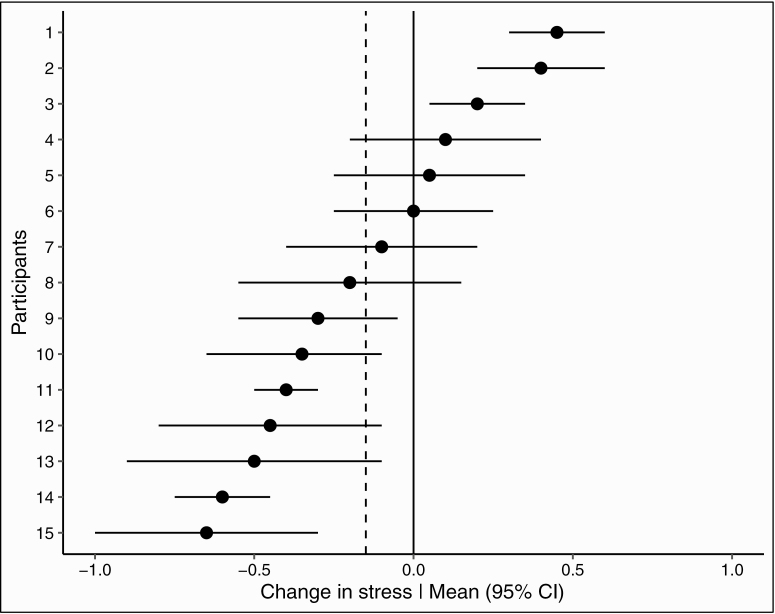
Simulated forest plot of within-person associations between physical activity and stress levels the following day, inspired from the results obtained in [35]. Effect sizes with 95% confidence intervals for each participant are presented on the *x*-axis. The dashed vertical line represents the average effect across participants. The forest plot of within-person associations allows the visualization of heterogeneity for a particular average effect. Here, physical activity is significantly associated with higher levels of stress for the first 3 participants (1–3); the association is nonsignificant for the next five participants (4–8); and physical activity is significantly associated with lower levels of stress for the remaining seven participants (9–15).

Adopting an idiographic approach to statistical modeling in behavior change research has several advantages. With regards to feasibility, when little is known about a complex phenomenon, the idiographic approach can result in more rapid, efficient, and cost-efficient learning that is directly applicable to and useful for the unit from whom the data were collected (e.g., a patient), compared with the traditional, nomothetic approach [35,36]. At the conceptual level, modeling individual trajectories, rather than averaging them, helps to better understand heterogeneity with regards to a particular behavioral influence or an intervention’s effectiveness [37,38]. Ultimately, the modeling of individual trajectories can help inform the development of highly tailored interventions, in line with the objectives of precision health [39,40] (e.g., identifying relevant, actionable variables to intervene on for a particular individual in a specific context). Finally, individual statistical modeling prevents potential fallacies of unfounded group-to-individual generalizability [15]. Illustrating this issue, a study investigating the daily relationships between physical activity and stress over a year shows that, on average, physical activity on a day was significantly associated with a reduction in stress at the end of the day [41]. However, at the individual level (i.e., using individual statistical modeling to unpack relationships for each participant), physical activity was significantly associated with stress for only 20% of the participants (15 of the 69 participants; a similar effect is illustrated in Fig. 2). Research adopting the idiographic approach has therefore stressed the importance of demonstrating consistency between group- and individual-level inferences [15].

Various visualization techniques and statistical options are available to model trajectories or explore a specific question for a single individual, although the particular analytic technique used largely depends on the study objective and the nature of the data [42–45]. Extensions of several statistical approaches, e.g., generalized linear mixed models, generalized additive mixed models, network analyses, or Bayesian analyses, can be applied to explore hypotheses at the individual level [46–48]). Slightly more complex and exploratory options are also available to identify a unique set of determinants/predictors for a specific behavior and individual (i.e., tailoring variable selection). For example, classification procedures, also referred to as machine learning methods (see [49]), have been used to predict smoking behavior [50], and system identification techniques, derived from the field of control systems engineering, have been used to identify factors associated with walking behavior [51]. It should also be noted that, often, idiographic and nomothetic approaches can be usefully combined; a nomothetic approach may be prioritized at the beginning of the data collection period, subsequently switching to idiographic modeling when a sufficient number of observations per participant becomes available [52].

### From narrative theoretical models to dynamic computational models

Clearly specifying beliefs, assumptions, hypotheses, and, by extension, theories about a given phenomenon is foundational for conducting rigorous, confirmatory science [53]. Returning to our point about complexity, experimental hypotheses and theories need to be specifiable in a way that matches the inherent complexity of the phenomenon under investigation. It has been argued that, to be complete, a theoretical model must contain four essential elements: *what* the key constructs are, *how* and *why* they are related, *where* and *when* they are applicable, and to *whom* [54]. Yet, in extant models of behavior change, three of these elements are typically under-specified: the temporal, contextual, and individual components [20].

As argued elsewhere [55–57], one way of addressing the complexity of behavior change theories is to use computational modeling, i.e., the translation of a model’s assumptions into mathematical equations. Computational modeling makes explicit the functional forms of the relationships between a model’s components and how these vary over well-specified time periods and scenarios representing, for example, different contexts (e.g., behavioral initiation vs. maintenance), the effects of exogenous variables (e.g., increases in atmospheric pollution), or baseline individual differences (e.g., age or gender). These scenarios can then be tested via computerized simulations, which provide multiple advantages. First, simulation models enable quick and inexpensive “sanity” checks on assumptions and beliefs. If results from the simulation appear wrong in some way to the scientist or its community, then the equations and hypotheses can be refined, refuted, or extended in a transparent manner [58]. Second, simulation studies enable the formalization of hypotheses that would be difficult to explore or visualize empirically due to practical or technical limitations, such as emergent properties of a system (see examples of agent-based models in public health [59]), or life-span analyses [60]. For example, computational modeling and simulation techniques have recently been used to understand how changes to a city’s infrastructure can lead to the nonlinear adoption of cycling behavior [61]. Finally, at the conceptual level, simulation helps to better formalize behavior change theories in terms of temporal, contextual, and individual aspects, which can ultimately help to disseminate theories to researchers, practitioners, and policy-makers, and generate ideas for empirical studies that challenge their assumption (computational modeling and associated simulation techniques are available in many standard data analytic tools, such as R, MatLab, or Python). As such, researchers interested in developing computational models can seek out the tools within the analytics environment they are most comfortable with. Also, the references above to different types of modeling and simulation techniques (e.g., fluid analogies, agent-based simulations, artificial neural networks) provide some initial examples for researchers to start using these tools.

Computational models and associated computerized simulations are still relatively under-used in the behavior change field. Through a literature search on PubMed, we identified articles revisiting classic health psychology models such as the Theory of Planned Behavior and Social Cognitive Theory via “artificial neural networks” and “fluid analogies and control systems principles” [62,63]. We also identified studies aiming to: quantify the effect of non-sugar-sweetened beverage placements in corner stores on adolescent purchasing behaviors via a “decision-analytic model” [64]; test which criteria influence agents’ impact in a social network intervention to increase physical activity in adolescents through “agent-based simulations” [65]; or predict change in body mass and composition during the course of a behavioral intervention for weight loss using a “dynamic model” inspired by control systems engineering methods [66]. More frequent use of computational modeling techniques could positively impact behavior change research in the near future by helping researchers to think dynamically and by better theorizing about how the context influences a particular behavior for a particular individual [57]. At the interventional level, computational models provide a direct foundation (i.e., mathematical algorithms) for building adaptive and personalized health behavior change interventions [67,68].

### From static to adaptive and continuous tuning interventions

The amounting evidence that health behaviors are complex suggests that adaptive and continuous “tuning” interventions are necessary for better supporting healthy behavioral choices over time and across contexts, similar to what a clinician or health coach would do in their practice [2,69]. The idea that tailored interventions are more likely to engender change at the individual level (as opposed to static or generic interventions) is not new; tailoring refers to a category of interventions which aim to “reach one specific individual, based on specific characteristics of that person that have been measured in a formal assessment” [70]. However, most tailored interventions so far have harnessed data from a single time point (e.g., demographic or psychological characteristics measured at baseline), or previous moderation analyses, to determine *what* or *how* content is delivered [71]. Recent methodological advancements are enabling far more precision in terms of the concept of tailoring and its automation via digital technologies.

Prior work has postulated distinctions between generic, targeted, and adaptive interventions [72,73]. In brief, while *generic interventions* do not include specific individualization components, *targeted interventions* support decision-making based on static information, such as demographics, personality traits, or physical fitness at the beginning of an intervention. *Adaptive interventions*, however, support dynamic decision-making over time with adaptation algorithms generated based on insights from prior individuals. Recently, based on the logical extreme for tailoring of supporting a specific individual and methodological advancements, a fourth intervention class has been proposed: *continuous tuning interventions* [2]. By continuous tuning, we mean interventions that use data about the individual for whom support is being provided to progressively refine and “tune” the intervention content, delivery feature(s) or timing to the idiosyncrasies of the individual, similar to individual psychotherapy or health coaching sessions delivered face-to-face by a trained professional, but with a potentially greater temporal precision afforded by digital technologies.

The key distinction between adaptive and continuous tuning interventions is how data are used for adaptation over time. Adaptive interventions are driven by pre-specified adaptation algorithms generated and evaluated based on the response of prior individuals, using study designs such as the “micro-randomized trial” (MRT; [74]) and main effect testing of the decision rules, time-invariant moderation or time-varying moderation. Continuous tuning interventions, however, include real-time optimization algorithms, which can further adjust intervention content or delivery aspects to the needs of a specific individual, using methods such as reinforcement learning [75], control systems engineering [67], and *N*-of-1 study designs [76].

On the one hand, adaptive interventions include, but are not limited to, “just-in-time adaptive interventions” (JITAIs), and aim to provide the right type and intensity of support to individuals at the right time ([69,77]). The “right” moment to intervene (also referred to as a “just-in-time state”) may, for example, be characterized by an individual’s vulnerability to engage in (or avoid) the target behavior, or their availability and openness to receive support at a particular point in time (e.g., depending on their current activity, upcoming schedule, or mental state). Similar to the majority of present-day tailored interventions, early adaptive interventions have relied on data from prior participants to determine *when* or *how* to intervene for the next group. Central to the distinction between adaptive and continuous tuning interventions is that adaptive interventions typically use data to make better adaptation algorithms for future individuals; however, the data from those using the intervention are not harnessed to further refine the intervention(s) for those people themselves.

On the other hand, continuous tuning interventions go a step farther than adaptive interventions by using data about the individual to further adjust the intervention to that specific person [78]. A good analogy for a continuous tuning intervention is a health coach. Relevant to continuous tuning, a good health coach has several attributes: (i) a clear sense of “meaningful” behavioral targets, such as the current national physical activity guideline; (ii) the ability to actively monitor a person’s behavior; (iii) awareness of a person’s broader life circumstances and how those circumstances influence each person’s behavior; (iv) a repertoire of evidence-based behavior change techniques that can be used and adapted to each person; and (v) the capacity to learn about a person and continuously adjust (or “tune”) the type or manner in which support is delivered based on each person’s changing needs. New methods and technologies enable all five of these attributes to occur in an automated fashion or to be used in partnership with a human interventionist, with the fifth point the key distinguishing factor between adaptive and continuous tuning interventions. To illustrate, we will describe in greater detail the use of “controllers” from control systems engineering.

Control systems engineering is concerned with the modeling and control of dynamically changing systems. The term “controller” is used to denote the series of mathematical equations that are used to monitor, make predictions for, and control dynamic systems (e.g., a patient). Controllers can be both model-based and model-free [67,79]: model-based controllers use dynamic computational models as inputs (described in the previous section, see also [69]). There is hence a clear link between individual statistical and computational modeling, and continuous tuning interventions. Control systems are ubiquitous in our everyday lives (e.g., thermostats, insulin pumps) and are designed to achieve and maintain a particular goal state or set point (e.g., a specific level of daily physical activity). They achieve this by (i) sampling data at a suitably high resolution to identify any discrepancies between the person’s behavior (e.g., 6,000 steps/day) and the desired goal (e.g., 10,000 steps/day) and (ii) adjusting the timing, content, and dosage of interventions according to the feedback (e.g., daily monitoring of physical activity levels). As the effect of any input may be context-dependent, advanced controllers take account of past states and simulate what may happen during future states in order to select the most appropriate input at a given moment in time. The discrepancy between a controller’s prediction and a person’s actual behavioral response is incorporated into future decisions. For example, if a person starts being less responsive to suggested physical activity goals, the controller may adjust the goal or increase the reward a person will receive for meeting a goal to increase their motivation to strive toward it. If that does not work, the controller may adjust feedback after not meeting goals towards more actively using implementation intentions or education. The system will monitor how a person responds to each of these variations and will systematically use approaches that the person is more responsive to (for an example, see [67]).

This type of intervention goes beyond adaptation based on prior data to continuous tuning via the controller actively tuning and adjusting how it provides support dynamically based on how the person responds over time in different contexts. As part of the control systems approach, the “control optimization trial” (COT) aims to combine idiographic, observational data (i.e., observing and modeling within-person dynamics of health behaviors over time) with the development and optimization of continuous tuning interventions [67]. Although no published examples exist in the behavior change field, Hekler et al. have recently secured funds to conduct a COT to support the perpetual adaptation of the JustWalk app, which aims to promote physical activity.

## CONCLUSION

As many health behaviors are dynamic, multicausal, and manifest idiosyncratically, the present article argues that health behavior change research should progressively transition from low- to high-resolution behavioral assessments, from group-only to group and individual statistical inference, from narrative theoretical models to computational models, and from static to adaptive and continuous tuning interventions (see Table 2for definitions of the key terms used in the article). This approach is aligned with the precision medicine initiative, which aims to develop preventive strategies that better take individual variability into account [2].

**Table 2:** Glossary of key terms (methods or approaches) presented in the article

Key terms	Possible definition
Ecological momentary assessment (EMA); experience sampling method (ESM)	The repeated measurement of relevant behaviors and their determinants in or near real-time via smartphones or handheld devices.
Measurement burst design	Widely spaced successions of short EMA/ESM periods, stretching over longer time periods (e.g., months, years).
Digital phenotyping	The use of sensors and traces gathered passively via the use of digital tools and social media to infer psychological and behavioral constructs.
Idiographic vs. nomothetic approaches	The idiographic approach aims to model intra-individual data, one participant at a time (i.e., “*N*-of-1” study). The nomothetic approach uses group-level data to model between-subjects associations and intervention effects.
Computational modeling	The translation of theoretical assumptions into mathematical equations and the manipulation of variables via simulations in statistical environments.
Continuous tuning interventions	A type of intervention which uses real-time optimization algorithms to adjust intervention content or delivery aspects to the needs of a specific individual, using methods such as reinforcement learning, control systems engineering, and *N*-of-1 study designs.
Just-in-time adaptive interventions (JITAIs)	A type of adaptive intervention which aims to provide, mainly via digital tools (e.g., smartphones), the right type and intensity of support to individuals at the right time.
Micro-randomized trial (MRT)	A type of study design, invented to facilitate the optimization and evaluation of JITAIs. The MRT involves the randomization of intervention options at multiple (often hundreds) of decision points over a pre-specified trial period and enables the assessment of main effects of intervention components in addition to time-invariant and time-varying moderation effects.
Control systems engineering	A branch of engineering concerned with the modeling and control of dynamically changing systems, such as human behavior.
Controller	An umbrella term used to denote the series of mathematical equations that are used to control dynamic systems.
Control optimization trial (COT)	A type of study design which uses control systems engineering methods to support individual tailoring variable selection and the optimization of decision rules (i.e., controllers) to support the perpetual adaptation of an intervention.

The utilization of these methods and approaches also come with specific challenges. To mention a few: (i) enhanced technical, statistical, and mathematical skills will be required to manage long time-series, model complex non-linear change, and develop mathematical equations underpinning computational models (see [80,81]); (ii) ethical issues relating to the tracking and manipulation of sensitive, personal data (e.g., GPS-derived, biomedical or social media data; see [82,83]); (iii) an increase in participant burden and potential lack of engagement with high-resolution measures, especially when participants are asked to actively complete questionnaires or cognitive tests several times per day per week (see [84,85]); and (iv) an increasing ecological impact of the transition from low- to high-technological research practices in public health (e.g., direct and indirect pollution engendered by the production and utilization of information and communication technologies such as smartphones and wearables; see [86,87]).

Although these challenges have no simple solutions, the development of highly interdisciplinary collaborations will be necessary to ensure appropriate transition toward precision behavior science. If successfully implemented, the four recommendations made in this article have the potential to greatly improve our current public health and behavior change practices in the near future.
